# Knowledge and attitudes towards and prevalence of overweight and obesity among undergraduate students at Universiti Kuala Lumpur Royal College of Medicine: A cross-sectional study

**DOI:** 10.51866/oa.582

**Published:** 2024-06-17

**Authors:** Khairul Najmi Wafdi Khairul Azhan, Aina Amanina Abdul Jalil, Syarifah Syamimi Putri Adiba Syed Putera

**Affiliations:** 1 BPharm, MClin Pharm, Faculty of Pharmacy and Health Sciences, Royal College of Medicine Perak, Universiti Kuala Lumpur, Ipoh, Perak, Malaysia. Email: aina.amanina@unikl.edu.my; 2 BPharm, Faculty of Pharmacy and Health Sciences, Royal College of Medicine Perak, Universiti Kuala Lumpur (RCMP UniKL), Ipoh, Perak, Malaysia.; 3 BPharm, MClin Pharm, Faculty of Pharmacy and Health Sciences, Royal College of Medicine Perak, Universiti Kuala Lumpur (RCMP UniKL), Ipoh, Perak, Malaysia.

**Keywords:** Knowledge, Attitudes, Prevalence, Undergraduate, Obesity

## Abstract

**Introduction:**

Overweight and obesity pose significant health risks, affecting social and economic well-being and potentially hindering mental health and learning outcomes. This study investigated the knowledge, attitudes, and prevalence of overweight and obesity among undergraduate students at Universiti Kuala Lumpur Royal College of Medicine (UniKL RCMP), Malaysia.

**Methods:**

A total of 351 UniKL RCMP undergraduate students participated in this study. Descriptive analysis was used to evaluate the sociodemographic and educational profiles of respondents. The chi-square test was conducted to identify the relationship between the sociodemographic and educational profiles and the level of knowledge and attitudes towards overweight and obesity.

**Results:**

The prevalence of overweight and obesity was 25.4%. The majority of the respondents possessed a good level of knowledge regarding overweight and obesity (n=316, 90%). The respondents’ programme was significantly associated with their level of knowledge (P=0.001).

**Conclusion:**

This study provides important information to university authorities, students, lecturers, parents and other stakeholders in education and health sectors about the impacts of overweight and obesity on the physical, social and academic welfare of university students.

## Introduction

Recently, the prevalence of obesity and overweight among adults has significantly increased.^1^ Overweight and obesity are characterised as abnormal or excessive fat accumulation that could be harmful to human health.^2^ Anthropometric indices including body mass index (BMI) and total body fat percentage are widely used for assessing the physical well-being of young adults. BMI is calculated as weight in kilograms divided by the square of height in metres. BMI is the most useful index in assessing obesity and overweight, as it is measured consistently for both sexes and all age groups. In adults, the BMI indicating overweight is ≥25 kg/m^2^, while that indicating obesity is ≥30 kg/m^2^. A systematic review and meta-analysis conducted in 2021 showed that obese adults had up to 32% higher risk of depression than underweight adults.^3^

In the National Health and Morbidity Survey in 2019,^4^ 30.4% of adults were overweight, and 19.7% were obese, consistent with the increasing prevalence of overweight and obesity since the 1960s. In 2016, over 1.9 billion people aged ≥18 years were overweight, and over 650 million adults were obese.^5^ Overall, 39% of adults aged ≥18 years (39% of men and 40% of women) were overweight, and 13% were obese. The worldwide prevalence of obesity tripled from 1975 to 2016.^2^ The prevalence of obesity, especially among urban people, has reached epidemic levels in several developing nations including Malaysia.^3^ Over the past 20 years, Malaysia has been reported to have a high adult obesity rate, along with other Southeast Asian nations.^6^

Globally, obesity has become a growing public health concern and is common among university students. Some researchers have found that BMI is a good predictor of physical function, sleep quality and smoking habits among university students.^7^ These researchers have concluded that women tend to be concerned about their body weight: They consume more vegetables and fruits, exercise frequently and maintain a lower daily caloric intake.^8^ The use of digital media devices has also contributed to obesity since people of all ages have been relying more heavily on them. It is likely that the frequent use of these devices is directly related to a reduction of daily activities and the practice of a sedentary lifestyle.^9^ Obesity and overweight are also linked to psychosocial issues not only among adults but also among university students. Many university students experience anxiety and depression as they worry about their appearances and insecurities.^10^

Healthcare providers, such as doctors, pharmacists and nurses, play an important role in providing education to the public about the hazards of obesity and offering appropriate advice on how to maintain body weight within the normal range. This study aimed to determine the prevalence, knowledge and attitudes towards overweight and obesity among undergraduate students at Universiti Kuala Lumpur Royal College of Medicine (UniKL RCMP). Although some studies have evaluated the knowledge and attitudes towards obesity and overweight in Malaysia, there is limited evidence on the prevalence of obesity among undergraduate students.

### General objective

The general objective of the study was to explore UniKL RCMP undergraduate students’ knowledge and attitudes towards overweight and obesity.

### Specific objectives

The specific objectives were as follows:

i. To determine the prevalence of and knowledge and attitudes towards overweight and obesity among UniKL RCMP undergraduate students;
ii. To identify the relationship between the sociodemographic and educational profiles and the level of knowledge and attitudes towards overweight and obesity among UniKL RCMP undergraduate students.

## Methods

### Study design

A cross-sectional study was conducted using a voluntary anonymous self-administered online structured questionnaire. The questionnaire was widely distributed on Google Forms.

### Study population

UniKL RCMP undergraduate students from the following courses were included in this study: Bachelor of Medicine and Bachelor of Surgery (MBBS), Bachelor of Nursing Science, Bachelor of Pharmacy, Bachelor of Pharmaceutical Technology, Bachelor of Physiotherapy, Diploma in Nursing, Diploma in Pharmacy, Diploma in Physiotherapy, Diploma in Medical Imaging and Foundation in Medical Sciences. Students who could not comprehend the English language and were unable to complete the survey were excluded from the study.

### Sample size calculation

The Raosoft® sample size calculator^11^ was used to calculate the sample size for this study. The sample size was calculated based on a confidence interval of 95%, a margin of error of 0.05 and a response distribution of 50% using the recommended formula in the Raosoft sample size calculator. The population size of UniKL RCMP undergraduate students was approximately 2670. Based on this estimated calculation, the minimum sample size needed and finally included in this study was 336.

### Research instrument and scoring method

The questionnaire comprised 32 questions divided into three different sections: Sections B, C and D. Section B assessed the sociodemographic and educational characteristics of respondents, such as programme, age, sex and year of study. This section also evaluated the prevalence of overweight and obesity based on BMI.

Sections C and D consisted of questions adapted with minor modifications from the study conducted by Reethesh et al. in 2019.^12^ These sections focused on the knowledge and attitudes towards overweight and obesity. The questions were scored on a 5-point Likert scale: ‘definitely’, ‘probably’, ‘probably not’, ‘definitely not’ and ‘do not know’. Section C contained 12 items that evaluated the knowledge regarding the risk factors and complications of obesity.

The scores for the overall knowledge and attitudes were categorised using Bloom’s cutoff point, wherein a total score of 80%–100% was considered to indicate good knowledge and attitudes; 60%–79%, moderate knowledge and attitudes; and <60%, poor knowledge and attitudes.^13^

### Data analysis

Data were recorded and analysed using the IBM SPSS Statistics for Windows, version 21 (IBM Corp., Armonk, N.Y., USA). Descriptive statistics such as frequencies, percentages, standard deviations (SDs) and means were used. A P-value of <0.05 was considered statistically significant. The chi-square test was performed to identify the relationship between the sociodemographic and educational profiles and the level of knowledge and attitudes towards overweight and obesity among respondents.

**Table 1:** Respondents’ sociodemographic and educational profiles (N=351).

Characteristic	Category	n	%
**Programme**	Bachelor of Medicine and Bachelor of Surgery	108	30.77
	Bachelor of Nursing Science	11	3.13
	Bachelor of Pharmacy	68	19.37
	Bachelor of Pharmaceutical Technology	20	5.69
	Bachelor of Physiotherapy	24	6.84
	Diploma in Nursing	15	4.27
	Diploma in Pharmacy	51	14.53
	Diploma in Physiotherapy	25	7.12
	Diploma in Medical Imaging	23	6.55
	Foundation in Medical Sciences	6	1.7
Age	≤20 years	144	41.03
	21–25 years	202	57.53
	26–30 years	5	1.42
	≥31 years	0	0
**Sex**	Male	66	18.8
	Female	285	81.2
**Year of study**	1	139	39.6
	2	113	32.19
	3	64	18.23
	4	24	6.84
	5	11	3.13

## Results

### Sociodemographic and educational profiles

A total of 351 undergraduate students recruited from March to May 2023, with no exclusions due to missing data, participated in this study. The sociodemographic and educational characteristics showed a diverse distribution, with the majority (30.8%) of the respondents enrolled in the MBBS degree programme. Approximately 81.2% of the respondents were women, while 57.5% were aged 21–25 years. Most respondents were second-year students (39.6%), while only a minority (3.1%) were fifth-year students. The sociodemographic and educational characteristics of the respondents are summarised in Table 1.

### Prevalence of overweight and obesity

Among the 351 respondents, 87 (24.8%) and 2 (0.6%) were overweight and obese, respectively. Conversely, 206 (58.7%) were underweight, and 56 (16%) had a normal weight. The mean (±SD) prevalence of overweight and obesity among the respondents was 2.09±0.6 based on the formula by Alexander et al.^14^:


$$\text{Prevalence}=\frac{\text{Total number of overweight and obese respondents}}{\text{Size of population}}\times 100$$

The prevalence of overweight and obesity was 25.36%. Most respondents (n=206, 58.7%) were considered to have a normal weight.

### Knowledge regarding overweight and obesity

The majority of the respondents were aware of key concepts, with 71.5% recognising BMI as an indicator of obesity. Notably, 88.6% acknowledged the association between obesity and NCDs. The respondents demonstrated an understanding of specific risk factors such as cardiovascular implications of abdominal fat (49.9%) and the link between obesity and diabetes mellitus (70.9%). Additionally, the respondents recognised the influence of lifestyle factors on weight gain, including excessive sugar consumption (79.5%), sugary beverage intake (82.1%) and fried food consumption (82.6%). However, fewer participants were aware of the effectiveness of fasting and skipping meals for weight loss (30.2%). The mean (±SD) knowledge score of the respondents was 52.85±4.106. The respondents demonstrated a good level of knowledge regarding overweight and obesity based on a scoring rate of 88.08% (52.85/60x100). Table 2 outlines the distribution of respondents’ knowledge levels regarding overweight and obesity.

**Table 2:** Distribution of the respondents’ knowledge regarding overweight and obesity (N=351).

Level	n (%)	Total mean (standard deviation) score
Good	316 (90)	52.85 (4.106)
Moderate	33 (9.4)	
Poor	2 (0.6)	

### Attitudes towards overweight and obesity

Of the respondents, 61.5% thought they were not overweight, while 39.3% thought their current weight did not affect their health at all. Approximately 58.4% of the respondents strongly agreed with the importance of eating breakfast regularly for a healthy lifestyle, and 51% believed that eating small frequent meals is beneficial for weight loss. The respondents generally had positive attitudes about exercising, doing housework, climbing stairs and walking to nearby places. The mean attitude score was 51.84±6.56, indicating that 69.12% of the respondents (51.84/75x100) had a poor attitude towards overweight and obesity. Table 3 outlines the distribution of respondents’ attitudes towards overweight and obesity.

**Table 3:** Distribution of the respondents’ attitudes towards overweight and obesity (N=351).

Level	n (%)	Total mean (standard deviation) score
Good	316 (90)	52.85 (4.106)
Moderate	33 (9.4)	
Poor	2 (0.6)	

### Relationship between the sociodemographic and educational profiles and the level of knowledge and attitudes towards overweight and obesity

The respondents’ programme (P=0.001) was significantly associated with their level of knowledge. Conversely, the respondents’ age (P=0.340), sex (P=0.344) and year of study (P=0.119) were not significantly associated with their level of knowledge. Further, no significant association was noted between the respondents’ programme (P=0.438), age (P=0.757), sex (P=0.203), year of study (P=0.326) and attitudes towards overweight and obesity.

## Discussion

### Prevalence of overweight and obesity

This study demonstrated that there was a moderate prevalence of overweight and obesity among UniKL RCMP undergraduate students, with only 89 of the 351 respondents (25.4%) categorised as being overweight or obese. This finding is similar to the report by Deotale et al.^15^ wherein medical students in India showed a moderate prevalence of overweight and obesity. Another study conducted among medical students in Nerul, India, also found a moderate prevalence of overweight (n=184, 36.8%) and obesity (n=55, 11.1%). 16 However, a study conducted among medical students in Trivandrum, Kerala, showed that 176 (50.28%) of 350 students were overweight or obese; 147 (42%) had a normal weight; and 27 (7.7%) were underweight.^17^ In another study among medical students from Western Balkans,^18^ the prevalence of overweight was 12%, while that of obesity was 2.3%, which is lower than that in our study. Chauhan and Modi^19^ also reported a higher prevalence of overweight (n=67, 21.6%) and obesity (n=97, 31.3%) among MBBS students at the Himalayan Institute of Medical Sciences, Uttarakhand. In another study conducted among medical students in Malaysia, 14.8% were overweight, and 5.2% were obese.^20^ Conversely, Rai and Makaju^21^ reported a much lower prevalence of overweight among 75 (19.48%) out of 385 medical students. These variations highlight the diverse prevalence of overweight and obesity among different student populations.

### Knowledge regarding overweight and obesity

In this study, the respondents’ level of knowledge was categorised as good based on a scoring rate of 88.08%. The mean (±SD) knowledge score was 52.85±4.106. In the previous study by Xue et al.,^22^ which involved 1317 randomly selected students, respondents had poor knowledge of overweight and obesity. Most of them were unfamiliar with the facts and information regarding overweight and obesity. The findings of our study are almost in line with those of the study by Diao et al.,^23^ which found that most respondents (80%) had a good knowledge level about the causes and complications of obesity but a poor knowledge level regarding the association of obesity with bone, tumour and reproductive diseases. The study conducted by Laar et al.^24^ among university employees in Pakistan found that most respondents (n=116, 61.4%) agreed with physical activities and exercises being the ways to prevent obesity, consistent with our findings (n=273, 77.8%). A previous study performed in 2020 reported that the majority of Portuguese undergraduate students believed that excessive sugar consumption can lead to weight gain, which is similar to our findings wherein more than half of the respondents also believed the effect of excessive sugar consumption. This study indicated that respondents were aware that a frequent intake of fried foods can be a risk factor for obesity.^25^ The findings from a Mediterranean cohort also confirmed that the frequency of fried food intake (more than four times per week) can increase the risk of obesity.^26^

Cultural influences and societal norms can impact the understanding of weight-related issues. Studies conducted in regions such as Pakistan and Portugal reveal cultural beliefs and dietary habits that influence the knowledge and perceptions of respondents regarding the causes and consequences of obesity. Studies involving university students, such as the one conducted by Xue et al.,^22^ show varying levels of exposure to information about overweight and obesity, leading to differences in knowledge levels among students. The alignment of the findings with previous reports suggests a positive trend in the knowledge levels among respondents, particularly regarding the factors associated with obesity.

### Attitudes towards overweight and obesity

In the present study, the attitudes of the respondents towards overweight and obesity were moderate based on a scoring rate of 69.12%. However, the study conducted by Shahid et al.^27^ in Faisalabad, Pakistan, showed that 98% of medical students possessed a good attitude. This proportion is larger than that in our study (77.5%). Kumar and Hiremath^28^ also reported that the degree of attitude of their respondents from the College of Horticulture, Bengaluru, was good (84%). A study conducted among young adults in Kuala Lumpur showed that they had a positive attitude based on their confidence with their ideal weight (mean=3.778, SD=0.977) and satisfaction with their weight (mean=3.624, SD=1.179).^29^ In the present study, the respondents demonstrated a positive attitude towards a healthy lifestyle, consistent with the report by Abu et al.^30^

The educational background and exposure of the respondents in this study influenced their attitudes towards weight management. Young adults in Kuala Lumpur^29^ and adolescents in Turkey^30^ have benefitted from specific health educational programmes or cultural norms that promote positive attitudes towards weight management and healthy living. The findings suggest a need for continued efforts to promote positive attitudes and behaviours related to weight management and healthy living among students.

## Conclusion

In conclusion, UniKL RCMP undergraduate students have a good level of knowledge regarding overweight and obesity, a moderate degree of attitude towards overweight and obesity and a moderate prevalence of overweight and obesity. None of the sociodemographic and educational profiles, except the programme, were associated with the level of knowledge and attitudes towards overweight and obesity. This study provides important information to university authorities, students, lecturers, parents and other stakeholders in the education and health sectors about the impacts of overweight and obesity on the physical, social and academic welfare of university students.

## Data sharing statement

The raw data for this study will be made available upon request.

**How does this paper make a difference in general practice?**

- The findings can guide Universiti Kuala Lumpur Royal College of Medicine (UniKL RCMP) authorities to optimise students’ free time by focusing on sports and physical activity.
- The findings can act as key tools for measuring student healthcare readiness and identifying areas for improvement. As students consider future careers in the medical field, it is important to assess their knowledge of weight management to encourage healthy living.
- Strategic actions can be implemented based on the findings to increase student awareness and ensure that UniKL RCMP graduates enter their chosen profession well-prepared and knowledgeable.
